# High Serum Folate Concentration Is Associated with Better Lung Function in Male Chronic Obstructive Pulmonary Disease Patients Who Are Current Smokers: Analysis of Nationwide Population-Based Survey

**DOI:** 10.3390/nu12082219

**Published:** 2020-07-25

**Authors:** Taeyun Kim, Chul-Ho Oak, Mann-Hong Jung, Tae-Won Jang, Jehun Kim

**Affiliations:** 1 Division of Pulmonology, Department of Internal Medicine, The Armed Forces Goyang Hospital, Gyeonggi-Do 10271, Korea; jimsb89@naver.com; 2 Division of Pulmonology, Department of Internal Medicine, Kosin University Gospel Hospital, Kosin University College of Medicine, Busan 49267, Korea; oaks70@hanmail.net (C.-H.O.); jaymh@dreamwiz.com (M.-H.J.); jangtw22@hanmail.net (T.-W.J.)

**Keywords:** folate, COPD, lung function, spirometry, KNHANES

## Abstract

Folate, folic acid, has a role in mitigating inflammatory reactions in the human body. This study aimed to evaluate the association of serum folate levels with lung function in chronic obstructive pulmonary disease (COPD) patients. Of the 8149 participants of the 2016 Korean National Health and Nutrition Examination Survey (KNHANES), 311 subjects (192 males and 119 females) having COPD defined by the lower fifth percentile of the reference population were selected. Pearson’s correlation coefficient was used to investigate the relationship between serum folate level and lung function measurements. The association between the serum folate level and lung function in patients with COPD was evaluated using multivariable linear regression analysis after adjustment for age, sex, height, high sensitivity C-reactive protein, total calorie intake, residence, smoking status and smoking pack–years, education, and household income. The serum folate level showed a positive correlation with the predicted percentage of forced expiratory volume in one second (FEV_1_%). In males, a trend for a positive correlation with serum folate level was observed in predicted FEV_1_%, FEV_1_ value, predicted percentage of forced vital capacity (FVC%), FVC value, and peak expiratory flow (PEF). No significant correlation between the serum folate level and lung function in females was observed. In the multivariable linear regression model, the serum folate level was associated with an increase in predicted FEV1%, FEV_1_ value, predicted FVC%, FVC value, and PEF; however, the significance was only observed in males, especially among current smokers. High serum folate level was positively associated with lung function measurements in male COPD patients who were current smokers. Further longitudinal studies are needed to elucidate the underlying mechanisms.

## 1. Introduction

Chronic obstructive pulmonary disease (COPD) is a treatable and preventable disease that encompasses not only airway inflammation and limitation, but also the systemic consequences and comorbidities [1]. The strive to reduce the global disease burden of COPD has reached a trend for decline in age-standardized mortality rates, especially in Western countries [2]. However, in Asia, together with population aging and expansion, high rates of smoking, and air pollution, COPD will remain responsible for disability, high economic burden, and disease-related mortality [3]. According to the Global Burden of Disease Study, COPD is the third leading cause of death worldwide, and an estimated three million people die due to the disease [4]. Moreover, approximately 90% of death related to COPD occur in low- and middle-income countries [5] where nutritional deficits are prevalent. The management and prevention of COPD is an important part of controlling the disease burden.

Although smoking abstinence remains a critical part of COPD management, how other modifiable factors and lifestyle affect lung function in patients with COPD is less clear because of its multifactorial and heterogenic nature. Recently, several studies have examined the relationship between nutritional status and spirometric measurements in patients with COPD. The intake amount of thiamin, riboflavin, niacin, and vitamin C were related to an increased value of the predicted value of forced expiratory volume in one second (FEV_1_) in elderly COPD patients [6]. One pilot study in COPD patients showed that hyperhomocystenemia, which is a risk factor for cardiovascular disease, was associated with poor folate status, and a dietary folate supplement lowered the serum homocysteine (Hcy) level [7]. In another epidemiologic study, high folate intake was beneficial for chronic smokers through improving lung function [8]. In a small sample-size study in Japan, folate intake relieved breathlessness and ameliorated lung function in COPD patients [9].

Folate is one of the B vitamins, which is also known as folic acid or vitamin B9. Folate is essential for several biochemical pathways in humans, including the methylation of amino acids, DNA, and lipids, as well as hemoglobin synthesis, and fetal neural tube development [10]. Folate is widely distributed in foods such as dark green leafy vegetables, legumes, nuts and seeds, fruits, liver, and yeast. Concerning the Korean diet, kimchi, rice, mandarins, laver, and chicken eggs are the major sources of dietary folate [11]. Folate may improve vascular endothelial function and lower the risk of stroke and cardiovascular disease by lowering Hcy levels and the associated antioxidative properties [12]. In addition, inverse dose-dependent relationships were observed in the association between serum folate level and serum total Ig E, atopy, wheezing, and physician-diagnosed asthma [13].

Although folate supplementation and a high serum folate level showed a positive effect on health-related problems, there is a paucity of data regarding the association between serum folate level and lung function in COPD patients. Considering that folate plays a central role in the metabolism of Hcy, only a few studies have examined the relationship between the serum concentration of Hcy and the severity of COPD. The serum Hcy level was significantly higher in COPD patients with a predicted value of FEV_1_ <50% than in patients with ≥50% [14]. A recent meta-analysis suggested that a low serum Hcy level could be a risk factor for COPD. However, the study included only four studies in the final analysis, fewer subjects included in each study, and the sub-group analysis was not performed [15].

Despite the previous studies, the effect of serum folate level on lung function parameters in patients with COPD, to our knowledge, has not been established, especially in Asian countries, where dietary patterns are different from Western countries. Furthermore, since previous studies have evaluated these micronutrients using food frequency questionnaires, relatively few studies have assessed the serum concentration of folate. In this context, the present study aimed to investigate the association between serum folate level and lung function parameters in patients with COPD using the dataset of the Korean National Health and Nutrition Examination Surveys (KNHANES) from 2016.

## 2. Materials and Methods

### 2.1. Study Subjects

The dataset of KNHANES from 2016 was used. KNHANES was designed using a multistage complex sampling method to obtain a nationally representative sample from non-institutionalized Korean citizens. The subjects were selected by proportional allocation system sampling with multistage stratification based on age, sex, and geographical area. KNHANES includes laboratory results, physical examination, health-related interview, and nutritional questionnaires. This cross-sectional and population-based nutritional and health survey is annually conducted by the Division of Chronic Disease Surveillance under the Korea Centers for Disease Control and Prevention and the Korean Ministry of Health and Welfare. The detailed protocols of the survey are described in a previous study [16].

The 2016 KNHANES assessed the health and nutritional statuses of 8149 Koreans. Because pulmonary function testing (PFT) was performed among subjects aged over 40 years old, the data of 3444 (42.3%) adults were available. Of these adults, subjects with the value of FEV_1_ divided by forced vital capacity (FVC) below the lower limit of normal (LLN) of the reference population were selected due to the high probability of having COPD [17]. Then, the subjects with missing values in the interested variables and potential confounders were excluded. Subjects diagnosed with asthma by their physician or taking medication for asthma were also excluded. Finally, 311 subjects (192 males and 119 females) with COPD were included in the study.

### 2.2. Data Collection and Measurements

Laboratory samples were obtained in a mobile examination center (MEC) for the exclusive use of KNHANES, drawn by medical assistants, and transported to the Central Laboratory (NEODIN Medical Institute, Seoul, Korea). The serum folate level was determined by the chemiluminescent microparticle immunoassay method using ARCHITECT i4000Sr (Abbott Laboratories, Abbott Park, IL, USA). High sensitivity C-reactive protein (hs-CRP) level was determined by the immunoturbidimetry method using a Cobas (Roche Diagnostics, Basel, Switzerland).

PFT was performed by a trained medical technician and examined in the MEC. Spirometry was conducted using dry rolling seal spirometers (Model 2130; SensorMedics, Yorba Linda, CA, USA). The quality control and standardization was conducted based on the criteria of the American Thoracic Society and European Respiratory Society [18]. Subjects with FEV_1_/FVC% lower than LLN were considered to have COPD [17]. The indices of lung function in the subjects with COPD included FEV_1_ (Liter, L), predicted FEV_1_%, FVC (L), predicted FVC%, FEV_1_/FVC%, and peak expiratory flow (PEF, L/s). Subjects with COPD were further classified into three groups, based on the classification of the Global Initiative for Chronic Obstructive Lung Disease: mild (predicted FEV_1_% ≥ 80), moderate (80 > predicted FEV_1_% ≥ 50), and severe (50 > predicted FEV_1_%).

Smoking status was categorized into never, former, and current smokers, based on the National Health Interview Survey classification of Centers for Disease Control and Prevention. Never smoker was defined as anyone who had consumed less than 100 cigarettes or does not currently smoke. Former smoker was defined as anyone who had consumed more than 100 cigarettes in the past year but does not currently smoke. Current smoker was defined as anyone who consumed more than 100 cigarettes and currently smokes.

Anthropometric variables were measured by trained survey assistants. Body mass index (BMI) was calculated as the body weight (kg) divided by the squared height (m^2^). Height was measured to the nearest 0.1 cm, and weight was measured to the nearest 0.1 kg.

Sociodemographic characteristics, including residence, household income, and educational level, were obtained by self-administered questionnaires and face-to-face interviews. The residential area was categorized into rural and urban. Educational level was divided into three groups: middle school or lower, high school, and college or more. The household income level was calculated as the average income of all family members and divided into quartiles.

### 2.3. Statistical Analysis

KNHANES data were produced using multistage, stratified, and probability sampling. Therefore, all statistical analyses were performed under complex sample analyses in SPSS. Sampling weights were applied for study subjects to represent the non-institutionalized Korean population.

Categorical variables were presented as weighted percentages with standard error (SE). Continuous variables were presented as the mean value with SE and the median value with the interquartile range (IQR).

Pearson’s correlation coefficient was calculated to measure the relationship between the age, hs-CRP, serum folate level, predicted FEV_1_%, FEV_1_ value, predicted FVC%, FVC value, and PEF. The serum folate level was log-transformed because its distribution violated the assumption of normality. The multivariable linear regression model was used to determine the association between serum folate level and lung function. In the multivariable analysis, age, sex, height, hs-CRP, total calorie intake, residence area, smoking status and smoking pack–years, educational level, and household income were included. Before the analysis, multicollinearity tests were performed to identify any inter-correlation between potential confounders. Coefficient levels with 95% confidence interval (CI) were estimated. Subgroup analyses were performed to identify any differences between males and females and between current, former, and never smokers. However, because there were only 22 never smokers among the male COPD patients and 13 current and two former smokers among the female COPD patients, we only performed a subgroup analysis among the current and former smokers in male COPD patients.

All statistical analyses were performed using IBM SPSS Statistics for Windows, version 25.0 (IBM Corp., Armonk, New York, NY, USA). *p*-values of less than 0.05 were considered statistically significant.

### 2.4. Ethical Approval

The study protocol was approved by the Institutional Review Board of the Kosin University Gospel Hospital (KUGH no. 2020-05-046). This study was conducted following the Declaration of Helsinki. All study procedures were following STROBE guidelines. Written informed consent was obtained from all subjects before participating in the survey.

## 3. Results

General clinical characteristics of the study subjects are presented in Table 1 (Table S1 for unweighted characteristics). Subjects with mild airflow limitation were younger than those with moderate and severe airflow limitation. Household income levels were higher in the mild group than in the moderate and severe groups. The mean serum folate levels were 4.86, 6.37, and 6.86 ng/mL in the severe group, moderate group, and mild group, respectively. The median serum folate levels were 3.50, 6.00, and 6.65 ng/mL in the severe group, moderate group, and mild group, respectively.

The correlations between age, serum folate level, hs-CRP, and lung function parameters in COPD patients are presented in Table 2. Serum retinol level was positively correlated with the predicted FEV_1_% (*r* = 0.172, *p*-value = 0.048).

The associations between the serum folate level and the lung function parameters are presented in Table 3. After adjustment for multivariable factors, predicted FEV_1_%, FEV_1_ value, predicted FVC%, FVC value, and PEF were positively associated with the serum folate level. In males, all lung function parameters were positively associated with serum folate level, and the significance remained after multivariable adjustment. However, in females, no significant association between the serum folate level and lung function parameters was observed.

**Figure 1:**
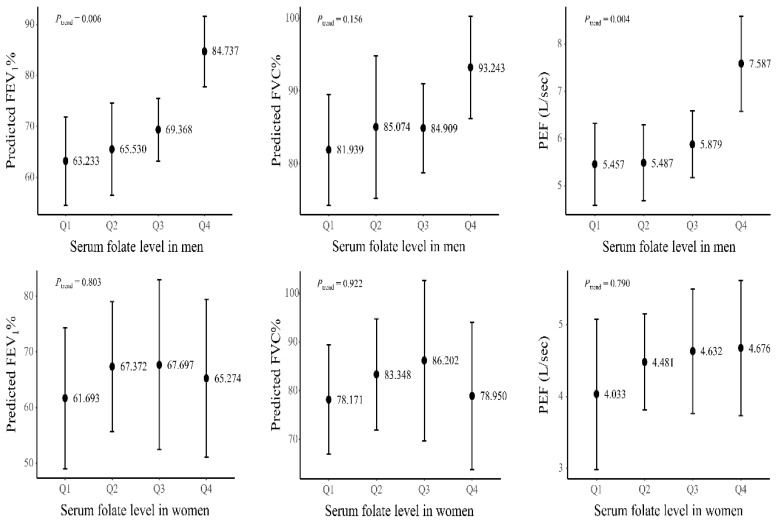
Association between the quartiles of serum folate level and predicted FEV_1_% and predicted FVC% with standard error according to the gender in patients with COPD. COPD, chronic obstructive pulmonary disease; hs-CRP, high sensitivity C-reactive protein; FEV_1_, forced expiratory volume in one second; FVC, forced vital capacity.

Figure 1 presents the association between the quartile of the serum folate level and the estimated lung function measurements with standard error when adjusted for multivariable factors (i.e., age, height, hs-CRP, total calorie intake, residence area, smoking status and smoking pack–years, educational level, and household income). In males, the serum folate level showed a positive relationship with the predicted FEV1% and predicted FVC%. On the contrary, no significant dose–response association was observed in females.

Table 4 shows the association between the serum folate level and the lung function parameters according to the smoking status in male COPD patients. In male current smokers, predicted FEV_1_%, FEV_1_ value_,_ predicted FVC%, FVC value, and the PEF showed a positive association with the serum folate level. However, there was no significant association between the serum folate level and the spirometric results among male former smokers.

Tables S2 and S3 present the correlation between the serum folate level, age, hs-CRP, and lung function parameters in males and females, respectively. Although the serum folate level showed a trend for positive association with lung function parameters in males, no significant correlations were observed in females.

## 4. Discussion

In this cross-sectional study, we found that the serum folate level was positively associated with several lung function parameters (i.e., predicted FEV_1_%, FEV_1_ value, predicted FVC%, FVC value, and PEF), especially in male current smokers. Although serum folate levels in male COPD patients were within the reference range [19], the lung function measurements were proportional to serum folate levels. In Korea, to our knowledge, this is the first population-based study investigating the relationship between the serum folate level and lung function measurements in patients with COPD. Our results are in line with several studies highlighting the beneficial aspects of this micronutrient. Because serum folate levels are responsive to dietary folate supplement [20], a fortified folate diet may be related to lung function improvement in patients with COPD, especially in male current smokers.

The serum folate level was not associated with lung function parameters in male COPD patients who were former smokers and female COPD patients. There are two possible explanations. First, the high proportion of smokers in males may affect this difference. The majority of male COPD patients (total 88.5%, former 50.8%, and current 37.7%) had experience of smoking, while only 12.7% of female COPD patients (former 1.7% and current 11.0%) were exposed to smoking. A previous study suggested that tobacco smoking may be attributable to reduced serum folate concentration [21]. Folate-dependent metabolism can be influenced by chemical components found in cigarette smoking, and consequently, folate transforms into inactive forms when exposed to smoking [22]. However, even after adjusting for smoking status and smoking pack–years, the association between the serum folate level and lung function remained statistically significant in males. The significance was intensified through adjustment for multivariable. Moreover, in our study, a subgroup analysis in male COPD patients according to the smoking status showed that significant relationships between the serum folate level and lung function were only found in current smokers. This relationship might be related to serum folate characteristics that reflect short term folate exposure [23]. Hirayama et al. found a similar association to our result, supporting that folate might be beneficial in male COPD patients, although the mechanisms are unclear [9]. Second, when given the same amount of folate supplement, subsequent serum folate levels could be different between males and females. Males need more folate supplementation than females to attain the same serum folate concentration because lean body weight is higher in males than in females [24].

This study evaluated the association between folate level and lung function by measuring the serum folate concentration. Biochemical analysis might be a more accurate way to assess the vitamin status than face-to-face questionnaires, although integrating both laboratory sampling and diet surveys are appropriate. Shvestov et al. suggested that 2–3 recurrent blood samplings could improve the correlation between serum micronutrient level and the true value within 20%, and four or more recurrent measurements could improve the correlation within 10% [25]. Food frequency questionnaires and 24 h recall surveys are commonly used methodologies to aggregate dietary intake data. However, study subjects do not completely remember the specific foods consumed, changing records in a timely manner, and inaccurately estimate food size [26]. Thus, day-to-day variability and recall bias can occur. We could not calculate the correlation between serum folate level and dietary folate intake since the information regarding the folate intake amount was not available in the 2016 KNHANES database.

Before applying our results to general COPD patients, the characteristics and the difference of the Korean diet from other countries should be considered. The Korean diet pattern is mainly composed of high carbohydrate, low fat, and abundant vegetables, and about 50% of adults follow the diet [27]. The common traditional Korean diet setting consists of rice, soup, kimchi, either cooked or raw vegetables, fermented beans, grilled fish or meat, and dry-preserved dishes [27]. The Korean diet group, compared to control, was related to a decrease in glycated hemoglobin level, triglyceride level, and blood pressure [27,28]. In respect of folate intake, *baechukimchi* (Chinese style cabbage kimchi), rice, spinach, eggs, and laver were the major source of this micronutrient for Koreans [29].

The association between folate and inflammation could partly explain the observed findings. First, the antioxidant activity of folate may have mitigated the airway and systemic inflammatory reactions of COPD patients. In a previous study, folate showed a capacity to reduce ferric cations, and other soluble vitamins (thiamine and pyridoxine) showed no ability to reduce Fe^3+^ [30]. Nakano et al. found a direct antioxidative effect of folate on the free-radical-induced oxidation of human low-density lipoprotein in an experimental model, suggesting the free-radical scavenging activity of folate [31]. A more recent in vivo and in vitro study similarly demonstrated the antioxidative capacity of folate by evaluating oxidative stress-induced apoptosis and the reactive oxygen species level [32]. This susceptibility of folate to oxidative stress could contribute to lowering the airway inflammation in COPD patients, as an airway inflammatory response is involved in the pathogenesis of COPD [33] and patients with an elevated inflammatory burden usually experience a rapid decline in lung function, increase in symptoms, and frequent exacerbations [34].

Second, high serum folate levels could indirectly affect inflammation by altering serum Hcy concentration in an inverse direction [7], which leads to lung function improvement in COPD patients. In male smokers, high serum Hcy level was an independent predictor for the rapid decline of predicted FEV_1_% and predicted FVC% [35]. Seemungal et al. demonstrated that elevated Hcy levels were inversely correlated to predicted FEV1% in COPD patients [14]. In this study, hs-CRP level was also higher in COPD patients than in the control group, suggesting that high serum Hcy levels may partially contribute to inflammation in COPD patients [14]. Our results also indicate that the hs-CRP level tends to be higher in patients with severe airflow limitation than those with mild airflow limitation. Considering that COPD is both an airway and systemic inflammatory disease, high serum folate levels may attenuate inflammation in patients with COPD and improve lung function.

Our study has several limitations. First, as the cross-sectional design could not address temporality issues, caution is needed when interpreting the results. The possibility of reverse causality should be considered. Second, we could not include dietary folate intake in the analysis due to a lack of information in the KNHANES database. Third, although the present study considered several potential confounding variables, other factors, such as the use of a bronchodilator, may affect the statistical significance. Fourth, because the serum folate level reflects the short-term folate status, the observed association in male COPD patients who are current smokers needs to be viewed with caution, and the level of accuracy and reliability of the chemical assays should be considered [23]. Fifth, although there are panels of markers for oxidative stress and inflammation, the KNHANES does not assess the biomarkers used to investigate inflammatory processes in the human body. Thus, further studies regarding the underlying mechanisms of the role of folate on the relationship between oxidative stress and COPD are warranted. Sixth, in the present study, the sample size of COPD patients included in the analysis was small. Despite these limitations, major strengths of this study are the robust adjustment for potential confounders related to the serum folate level and lung function parameters and reliable measurements of micronutrients using subjects’ serum samples. Nonetheless, further interventional studies are needed to elucidate the mechanisms underlying the observed findings. The results would benefit from further studies on the mechanisms linking the serum folate level and airway inflammation, as well as the quantitative conceptualization of the impact of a folate-rich diet on COPD symptom progression and morbidity/mortality.

## 5. Conclusions

In male COPD patients who are current smokers, high serum folate level was significantly associated with better lung function (i.e., predicted FEV_1_%, FEV_1_ value, predicted FVC%, FVC value, and PEF). The observed findings might be related to the antioxidative property of folate, which may alleviate the airway and systemic inflammatory reaction in male COPD patients who are current smokers. Based on the results of this study, future additional studies should investigate the longitudinal effect of folate on lung function in both Korean and other Asian populations to clarify the causal relationship between this nutrient and spirometry results.

## Figures and Tables

**Table 1:** Clinical characteristics of the study subjects according to the airflow limitation severity.

	Mild (*n* = 125)	Moderate (*n* = 163)	Severe (*n* = 23)	*p*-Value
**Population size**	1,012,555	1,237,035	164,374	
**Sex**				0.166
Men	59.4 (4.6)	65.4 (4.3)	81.3 (8.3)	
Women	40.6 (4.6)	34.6 (4.3)	18.7 (8.3)	
**Age groups**				<0.001 *,†
40–49	41.6 (4.9)	20.2 (4.0)	6.2 (6.0)	
50–59	24.6 (4.6)	27.8 (4.3)	13.4 (7.8)	
60–69	18.3 (3.5)	26.4 (4.1)	18.5 (9.6)	
≤70	15.4 (3.5)	25.6 (3.9)	61.8 (12.0)	
**Height (m)**	1.65 (0.0)	1.63 (0.0)	1.64 (0.0)	0.313
**Body mass index (kg/m^2^)**	23.6 (0.3)	23.4 (0.4)	22.7 (0.9)	0.660
**Residence**				0.030
Rural	15.4 (3.9)	24.7 (5.4)	35.4 (11.7)	
Urban	84.6 (3.9)	75.3 (5.4)	64.6 (11.7)	
**Smoking status**				0.196
Never	47.2 (4.8)	33.6 (4.2)	22.4 (9.0)	
Former	26.7 (4.4)	31.3 (4.4)	35.5 (11.6)	
Current	26.1 (5.6)	35.1 (4.7)	42.1 (12.9)	
**Smoking Pack**–**Years** §	26.2 (2.1)	31.1 (3.4)	34.0 (5.2)	0.250
**Education**				0.085
Middle school or less	32.7 (4.7)	38.3 (4.9)	66.8 (10.9)	
High school	37.7 (4.7)	32.3 (4.7)	23.1 (9.6)	
College or more	29.6 (4.1)	29.4 (4.3)	10.1 (6.1)	
**Household income**				0.002 †, ‡
Lowest	18.4 (3.7)	24.9 (4.2)	59.8 (11.9)	
Lower middle	19.0 (4.2)	27.7 (5.1)	26.6 (11.1)	
Higher middle	26.0 (4.6)	18.6 (3.9)	3.5 (3.5)	
Highest	36.7 (5.3)	28.8 (4.6)	10.1 (6.1)	
**Hs-CRP (mg/L)**				
mean (SE)	0.94 (0.2)	1.72 (0.4)	1.68 (0.4)	0.073
median (IQR)	0.46 (0.30–0.74)	0.80 (0.49–1.62)	1.50 (0.54–2.30)	
**Total calorie intake (kcal/day)**				0.485
mean (SE)	1925.4 (90.4)	2004.8 (76.3)	1769.6 (229.8)	
median (IQR)	1775.3 (1298.2–2476.5)	1970.5 (1466.7–2525.7)	1527.4 (861.8–2153.8)	
**Serum Folate level (ng/mL)**				0.124
mean (SE)	6.86 (0.5)	6.37 (0.5)	4.86 (0.8)	
median (IQR)	6.65 (4.58–9.43)	6.00 (4.40–9.13)	3.50 (3.10–4.70)	

Data are presented as the weighted mean (SE) and median (IQR) for continuous variables or weighted percentage (SE) for categorical variables, unless otherwise stated. The degree of airflow limitation was defined based on the Global Initiative for Chronic Obstructive Lung Disease (GOLD) report: mild (predicted FEV_1_% ≥ 80), moderate (80 > predicted FEV_1_% ≥ 50), and severe (50 > predicted FEV_1_%). *p*-value < 0.017 was considered significantly different between: * mild vs. moderate, † mild vs. severe, ‡ moderate vs. severe. § Smoking pack–years were calculated only among former and current smokers. Hs-CRP, high sensitivity C-reactive protein; FEV_1_, forced expiratory volume in 1 s.

**Table 2:** Correlation between the age, serum folate level, hs-CRP, and the lung function parameters in the patients with COPD using Pearson’s correlation analysis.

	Age (Years)	Serum Folate (ng/mL)	Hs-CRP (mg/L)	Predicted FEV_1_%	FEV_1_ Value (L)	Predicted FVC%	FVC Value (L)	PEF (L/s)
Age (years)	1	0.077 (0.378)	0.138 (**0.017**)	−0.345 (<**0.001**)	−0.640 (<**0.001**)	−0.400 (<**0.001**)	−0.464 (<**0.001**)	−0.476 (<**0.001**)
Serum folate (ng/mL)	0.077 (0.378)	1	0.032 (0.719)	0.172 (**0.048**)	−0.022 (0.800)	0.138 (0.113)	−0.064 (0.467)	−0.001 (0.990)
Hs-CRP (mg/L)	0.138 (**0.017**)	0.032 (0.719)	1	−0.153 (**0.007**)	−0.147 (**0.011**)	−0.113 (0.050)	−0.091 (0.115)	−0.171 (**0.003**)
Predicted FEV_1_%	−0.345 (<**0.001**)	0.172 (**0.048**)	−0.153 (**0.007**)	1	0.713 (<**0.001**)	0.881 (<**0.001**)	0.539 (<**0.001**)	0.572 (<**0.001**)
FEV_1_ value (L)	−0.640 (<**0.001**)	−0.022 (0.800)	−0.147 (**0.011**)	0.713 (<**0.001**)	1	0.696 (<**0.001**)	0.920 (<**0.001**)	0.850 (<**0.001**)
Predicted FVC%	−0.400 (<**0.001**)	0.138 (0.113)	−0.113 (0.050)	0.881 (<**0.001**)	0.696 (<**0.001**)	1	0.657 (<**0.001**)	0.512 (<**0.001**)
FVC value (L)	−0.464 (<**0.001**)	−0.064 (0.467)	−0.091 (0.115)	0.539 (<**0.001**)	0.920 (<**0.001**)	0.657 (<**0.001**)	1	0.790 (<**0.001**)
PEF (L/s)	−0.476 (<**0.001**)	−0.001 (0.990)	−0.171 (**0.003**)	0.572 (<**0.001**)	0.850 (<**0.001**)	0.512 (<**0.001**)	0.790 (<**0.001**)	1

COPD, chronic obstructive lung disease; hs-CRP, high sensitivity C-reactive protein; FEV_1_, forced expiratory volume in 1 s, FVC, forced vital capacity; PEF, peak expiratory flow. Bold denotes a *p*-value < 0.05.

**Table 3:** Association of the serum folate level with lung function parameters in the patients with COPD using the linear regression model.

	Crude		Age and (Sex) Adjusted		Multivariable-Adjusted	
	β (95% CI)	*p*-Value	β (95% CI)	*p*-Value	β (95% CI)	*p*-Value
**Total (*n* = 311)**						
Predicted FEV_1_%	13.315 (−0.487, 27.118))	0.058	12.134 (−1.556, 25.824)	0.081	33.260 (17.051, 49.469)	<0.001
FEV_1_ value (L)	−0.153 (−0.789, 0.482)	0.631	0.466 (0.039, 0.893)	0.033	0.875 (0.320, 1.430)	0.004
Predicted FVC%	7.944 (−2.757, 18.644)	0.143	9.289 (−0.932, 19.510)	0.074	22.918 (6.874, 38.961)	0.007
FVC value (L)	−0.559 (−1.371, 0.252)	0.173	0.562 (0.052, 1.073)	0.031	0.893 (0.200, 1.587)	0.014
PEF (L/s)	−0.341 (−2.002, 1.321)	0.683	1.358 (−0.081, 2.796)	0.064	3.216 (1.751, 4.681)	<0.001
**Men (*n* = 192)**						
Predicted FEV_1_%	17.590 (−4.043, 39.224)	0.108	21.260 (2.318, 40.023)	0.029	33.560 (14.986, 52.135)	0.001
FEV_1_ value (L)	0.514 (−0.510, 1.537)	0.315	0.774 (0.152, 1.395)	0.016	0.855 (0.175, 1.535)	0.017
Predicted FVC%	12.050 (−3.519, 27.618)	0.125	17.506 (5.406, 29.606)	0.006	21.766 (2.790, 40.741)	0.027
FVC value (L)	0.597 (−0.472, 1.667)	0.264	0.932 (0.230, 1.634)	0.011	0.872 (0.0005, 1.744)	0.050
PEF (L/s)	1.720 (−0.854, 4.294)	0.184	2.019 (−0.143, 4.181)	0.066	3.040 (1.277, 4.804)	0.002
**Women (*n* = 119)**						
Predicted FEV_1_%	−4.154 (−23.515, 15.207)	0.662	−2.732 (−17.499, 12.035)	0.706	8.422 (−6.523, 23.366)	0.255
FEV_1_ value (L)	−0.446 (−1.264, 0.372)	0.272	−0.062 (−0.611, 0.487)	0.819	0.264 (−0.123, 0.652)	0.171
Predicted FVC%	−4.957 (−26.443, 16.529)	0.639	−3.156 (−20.043, 13.730)	0.704	9.877 (−6.439, 26.193)	0.222
FVC value (L)	−0.505 (−1.597, 0.586)	0.349	0.009 (−0.795, 0.813)	0.982	0.458 (−0.065, 0.980)	0.083
PEF (L/s)	−0.771 (−2.674, 1.132)	0.412	−0.118 (−1.839, 1.604)	0.889	1.000 (−0.600, 2.599)	0.208

Serum folate was log-transformed because of its left-skewed distribution. Multivariable-adjusted model considered age, sex, height, hs-CRP, total calorie intake, residence area, smoking status and smoking pack–years, educational level, and household income as covariates. COPD, chronic obstructive pulmonary disease; FEV_1_, forced expiratory volume in 1 s; FVC, forced vital capacity; PEF, peak expiratory flow; CI, confidence interval; hs-CRP, high sensitivity C-reactive protein.

**Table 4:** Association of the serum folate level with the lung function parameters according to the smoking status in male patients with COPD.

	Age and Height Adjusted		Multivariable-Adjusted	
	β (95% CI)	*p*-Value	β (95% CI)	*p*-Value
**Current smokers (*n* = 72)**				
Predicted FEV_1_%	24.174 (2.230, 46.118)	0.032	27.481 (5.971, 48.992)	0.014
FEV_1_ value (L)	0.726 (0.107, 1.345)	0.023	0.879 (0.287, 1.472)	0.005
Predicted FVC%	16.842 (3.153, 30.531)	0.017	26.347 (12.030, 40.665)	0.001
FVC value (L)	0.801 (0.186, 1.416)	0.012	1.149 (0.544, 1.753)	<0.001
PEF (L/s)	3.297 (0.764, 5.829)	0.012	4.443 (2.067, 6.819)	0.001
**Former smokers (*n* = 97)**				
Predicted FEV_1_%	19.148 (−15.692, 53.987)	0.272	−3.142 (−28.970, 22.685)	0.804
FEV_1_ value (L)	1.062 (−0.224, 2.347)	0.103	−0.062 (−0.922, 0.798)	0.883
Predicted FVC%	14.371 (−15.869, 44.611)	0.341	−7.326 (−31.575, 16.922)	0.539
FVC value (L)	1.215 (−0.332, 2.763)	0.120	−0.396 (−1.553, 0.760)	0.486
PEF (L/s)	1.940 (−1.948, 5.828)	0.318	−0.316 (−2.806, 2.174)	0.796

Serum folate was log-transformed because of its left-skewed distribution. Multivariable-adjusted model considered age, height, hs-CRP, total calorie intake, residence area, smoking status and smoking pack–years, educational level, and household income as covariates. COPD, chronic obstructive pulmonary disease; FEV_1_, forced expiratory volume in 1 s; FVC, forced vital capacity; PEF, peak expiratory flow; CI, confidence interval; hs-CRP, high sensitivity C-reactive protein.

## Supplementary Materials

The following are available online at https://www.mdpi.com/2072-6643/12/8/2219/s1, Table S1. Unweighted clinical characteristics of the study subjects according to airflow limitation severity. Table S2. The correlation between age, serum folate level, hs-CRP, and lung function parameters in male COPD using Pearson’s correlation analysis. Table S3. Correlation between age, serum folate level, hs-CRP, and lung function parameters in female COPD using Pearson’s correlation analysis.
