# Supplementary material for: High Serum Folate Concentration Is Associated with Better Lung Function in Male Chronic Obstructive Pulmonary Disease Patients Who Are Current Smokers: Analysis of Nationwide Population-Based Survey
Source: Nutrients. 2020 Jul 25;12(8):2219. doi: 10.3390/nu12082219 (PMC7468925; doi:10.3390/nu12082219)
Supplement: Supplementary file 1 [file nutrients-12-02219-s001.pdf]

Supplementary Table S1. Unweighted clinical characteristics of study subjects according to airflow limitation severity.

	Mild (n = 125)	Moderate (n = 163)	Severe (n = 23)	*P*-value
**Sex**				0.055
Men, n (%)	71 (56.8)	103 (63.2)	18 (78.3)	
Women, n (%)	54 (43.2)	60 (36.8)	5 (21.7)	
**Age groups**				0.051
40-49, n (%)	45 (36.0)	28 (17.2)	1 (4.3)	
50-59, n (%)	31 (24.8)	33 (20.2)	3 (13.0)	
60-69, n (%)	28 (22.4)	52 (31.9)	5 (21.7)	
≤70, n (%)	21 (16.8)	50 (30.7)	14 (60.9)	
**Height (m),** mean (SD)	1.64 (0.1)	1.63 (0.1)	1.65 (0.1)	0.356
**BMI (kg/m^2),** mean (SD)	23.6 (2.6)	23.3 (3.4)	23.6 (4.0)	0.594
**Residence**				0.048
Rural, n (%)	24 (19.2)	48 (29.4)	9 (39.1)	
Urban, n (%)	101 (80.8)	115 (70.6)	14 (60.9)	
**Smoking status**				0.055
Never, n (%)	61 (49.2)	58 (35.8)	6 (26.1)	
Former, n (%)	35 (28.2)	55 (34.0)	9 (39.1)	
Current, n (%)	28 (22.6)	49 (30.2)	8 (34.8)	
**Smoking Pack-years** §, mean (SD)	26.1 (16.5)	31.3 (25.8)	38.4 (18.8)	0.103
**Education**				0.459
Middle school or less, n (%)	48 (39.7)	69 (44.2)	14 (60.9)	
High school, n (%)	39 (32.2)	48 (30.8)	5 (21.7)	
College or more, n (%)	34 (28.1)	39 (25.0)	4 (17.4)	
**Household income**				0.001†
Lowest, n (%)	28 (20.8)	50 (30.9)	14 (60.9)	
Lower middle, n (%)	24 (19.2)	44 (27.2)	5 (21.7)	
Higher middle, n (%)	29 (23.2)	27 (16.7)	1 (4.3)	
Highest, n (%)	46 (36.8)	41 (25.3)	3 (13.0)	
**Hs-CRP (mg/L)**				
mean (SD)	0.93 (1.9)	1.67 (3.0)	1.67 (1.5)	0.044
median (IQR)	0.50 (0.30-0.76)	0.80 (0.42-1.68)	1.35 (0.63-2.11)	
**Total calorie intake (kcal/day)**				0.772
mean (SD)	1949.3 (828.5)	1991.7 (750.3)	1854.6 (1002.8)	
median (IQR)	1725.5 (1342.8-2393.9)	1929.8 (1435.9-2663.2)	1288.4 (829.6-2021.9)	
**Serum Folate level (ng/mL)**				0.199
mean (SD)	7.25 (3.3)	6.71 (3.4)	5.27 (3.1)	
median (IQR)	6.30 (4.55-9.40)	6.00 (4.40-9.20)	3.70 (3.15-4.68)	

The degree of airflow limitation was defined based on the Global Initiative for Chronic Obstructive Lung Disease (GOLD) report: mild (predicted FEV_1% ≥80), moderate (80> predicted FEV_1% ≥50), and severe (50> predicted FEV_1%).

P-value <0.017 was considered significantly different between: *mild vs. moderate, †mild vs. severe, ‡moderate vs. severe.

§Smoking pack-years were calculated only among former and current smokers.

Hs-CRP, high sensitivity C-reactive protein; BMI, body mass index; FEV_1, forced expiratory volume in 1 second; SD, standard deviation; IQR, interquartile range.

Supplementary Table S2. Correlation between age, serum folate level, hs-CRP, and lung function parameters in male COPD using Pearson's correlation analysis.

	Age (years)	Serum folate (ng/mL)	Hs-CRP (mg/L)	Predicted FEV_1%	Trough FEV_1 (L)	Predicted FVC%	Trough FVC (L)	PEF (L/sec)
Age (years)	1	0.124 (0.277)	0.097 (0.188)	-0.400 (<0.001)	-0.751 (<0.001)	-0.448 (<0.001)	-0.632 (<0.001)	-0.598 (<0.001)
Serum folate (ng/mL)	0.124 (0.277)	1	0.092 (0.420)	0.205 (0.070)	0.163 (0.152)	0.211 (0.062)	0.229 (0.042)	0.215 (0.057)
Hs-CRP (mg/L)	0.097 (0.188)	0.092 (0.420)	1	-0.225 (0.002)	-0.188 (0.010)	-0.157 (0.031)	-0.143 (0.050)	-0.219 (0.003)
Predicted FEV_1%	-0.400 (<0.001)	0.205 (0.070)	-0.225 (0.002)	1	0.836 (<0.001)	0.856 (<0.001)	0.741 (<0.001)	0.755 (<0.001)
Trough FEV_1 (L)	-0.751 (<0.001)	0.163 (0.152)	-0.188 (0.010)	0.836 (<0.001)	1	0.800 (<0.001)	0.923 (<0.001)	0.842 (<0.001)
Predicted FVC%	-0.448 (<0.001)	0.211 (0.062)	-0.157 (0.031)	0.856 (<0.001)	0.800 (<0.001)	1	0.882 (<0.001)	0.664 (<0.001)
Trough FVC (L)	-0.632 (<0.001)	0.229 (0.042)	-0.143 (0.050)	0.741 (<0.001)	0.923 (<0.001)	0.882 (<0.001)	1	0.743 (<0.001)
PEF (L/sec)	-0.598 (<0.001)	0.215 (0.057)	-0.219 (0.003)	0.755 (<0.001)	0.842 (<0.001)	0.664 (<0.001)	0.743 (<0.001)	1

COPD, chronic obstructive lung disease; hs-CRP, high sensitivity C-reactive protein; FEV_1, forced expiratory volume in 1 second, FVC, forced vital capacity; PEF, peak expiratory flow.

Supplementary Table S3. Correlation between age, serum folate level, hs-CRP, and lung function parameters in female COPD using Pearson's correlation analysis.

	Age (years)	Serum folate (ng/mL)	Hs-CRP (mg/L)	Predicted FEV_1%	Trough FEV_1 (L)	Predicted FVC%	Trough FVC (L)	PEF (L/sec)
Age (years)	1	0.137 (0.322)	0.189 (0.043)	-0.186 (0.042)	-0.693 (<0.001)	-0.275 (0.002)	-0.662 (<0.001)	-0.607 (<0.001)
Serum folate (ng/mL)	0.137 (0.322)	1	0.156 (0.259)	0.033 (0.812)	-0.098 (0.479)	-0.021 (0.882)	-0.115 (0.408)	-0.042 (0.765)
Hs-CRP (mg/L)	0.189 (0.043)	0.156 (0.259)	1	-0.026 (0.785)	-0.130 (0.165)	-0.032 (0.733)	-0.105 (0.263)	-0.187 (0.045)
Predicted FEV_1%	-0.186 (0.042)	0.033 (0.812)	-0.026 (0.785)	1	0.752 (<0.001)	0.918 (<0.001)	0.701 (<0.001)	0.588 (<0.001)
Trough FEV_1 (L)	-0.693 (<0.001)	-0.098 (0.479)	-0.130 (0.165)	0.752 (<0.001)	1	0.754 (<0.001)	0.965 (<0.001)	0.823 (<0.001)
Predicted FVC%	-0.275 (0.002)	-0.021 (0.882)	-0.032 (0.733)	0.918 (<0.001)	0.754 (<0.001)	1	0.795 (<0.001)	0.554 (<0.001)
Trough FVC (L)	-0.662 (<0.001)	-0.115 (0.408)	-0.105 (0.263)	0.701 (<0.001)	0.965 (<0.001)	0.795 (<0.001)	1	0.769 (<0.001)
PEF (L/sec)	-0.607 (<0.001)	-0.042 (0.765)	-0.187 (0.045)	0.588 (<0.001)	0.823 (<0.001)	0.554 (<0.001)	0.769 (<0.001)	1

COPD, chronic obstructive lung disease; hs-CRP, high sensitivity C-reactive protein; FEV_1, forced expiratory volume in 1 second, FVC, forced vital capacity; PEF, peak expiratory flow.
